# Psychiatry, racism and crime: the case of Christopher Clunis reconsidered

**DOI:** 10.3389/fpsyt.2024.1334020

**Published:** 2024-02-05

**Authors:** Ian Cummins

**Affiliations:** School of Health & Society, University of Salford, Salford, United Kingdom

**Keywords:** psychiatry, racism, deinstitutionalisation, representation, mental illness

## Abstract

In December 2022, the death of Christopher Clunis was made public. He had actually died in February 2021. Christopher Clunis was convicted of the manslaughter of a stranger, Jonathan Zito. He attacked Mr Zito at a train station. This paper will argue that this terrible event became a totemic symbol of the wider failings of the policy of community care. The image of Clunis being driven away from Court was repeatedly used in newspaper and other media reports as a reference point. The image reflects a number of long-standing traits in the representation of the “mentally ill.” These are combined with a racial stereotype of Black men. The paper examines historical representations of the mentally ill as a context for a discussion of the Clunis case. The paper uses the work of Stuart Hall as an analytical tool to examine the questions of race and representation, and the moral panic following failings of community care.

## Introduction

In December 2022, the death of Christopher Clunis was made public. He had actually died in February 2021. Christopher Clunis was convicted of the manslaughter of a stranger, Jonathan Zito. He attacked Mr Zito at a train station. This paper will argue that this terrible event became a totemic symbol of the wider failings of the policy of community care. The image of Clunis being driven away from Court was repeatedly used in newspaper and other media reports as a reference point. The image reflects a number of long-standing traits in the representation of the “*mentally ill.”* These are combined with a racial stereotype of Black men. The paper examines historical representations of the mentally ill as a context for a discussion of the Clunis case. The paper uses the work of Stuart Hall as an analytical tool to examine the questions of race and representation, and the moral panic following failings of community care.

## Christopher Clunis

Christopher Clunis was born in Londonin1963. He began studying for A-levels but then decided to pursue a career as jazz guitarist. He worked on cruise ships. However, from 1986 onwards, Clunis’s mental health deteriorated significantly. His parents had returned to Jamaica. Clunis was first admitted to a psychiatric there. In1987, Clunis returned to live in London. Between 1987 and the murder of Jonathan Zito in December 1992, Clunis was admitted to hospital on several occasions, usually for very short periods. When he was discharged from hospital, he often experienced homelessness. He never received the intensive support he needed to address his serious mental health and related problems. When he was acutely unwell, Clunis had a history of violence including threats and assaults on nursing staff.

## Representations of mental illness

Madness is a challenging and disturbing topic. It both attracts and repels. “*Madness”* and mental illness despite being at the marginalised have been major topics for painters, novelists, poets, dramatists, and other artists from across a range of disciplines (1). Mental illness and the response to it raises huge ethical and moral questions. As it is a core feature of the human condition, it is not surprising that it is a theme that artists and writers return to. Representations of madness and mental illness are very influential in not only the development of broader societal attitudes but also the response to individuals in crisis (1). For example, the 2019 film *The Joker* portrays psychosis using a series of stereotypes that negatively affect society’s attitude toward mental health (2).

There have been a series of campaigns that challenge the stigma that surrounds mental illness. These campaigns are often led by very high-profile celebrities but do not examine the underlying political economy of stigma and discrimination (3). These campaigns also focus on particular forms or expression of distress – for example work related stress and burnout. This is in no way to diminish the importance of these issues or the terrible impact that they can have on individuals and families. However, this focus alongside a concentration on a bio medical model of mental illness can have the unintended consequence of reinforcing the Othering of those experiencing severe mental health conditions such as psychosis.

Pre-existing social representations of the *‘other’* are immensely powerful in their ability to create a new identity for social categories (4). It is possible to trace the ways, in which, representations of the *mad* from the asylum era has followed those people into the community. For example, from the 1990s onwards a homeless mentally ill (black) man with all his belongings in a shopping trolley became a TV and film drama cliché, a signifier of gritty urban realism. The presence of individuals effectively abandoned by the social state, living on the margins of society is now an almost accepted feature of modern urban life. Representations of mental illness became inextricably linked to incidents of violent crime (5). Not all of these crimes were committed by people with any history of mental illness. Concern about issues of civil rights or dignity were pushed into the background. This is a modern recasting of deeply engrained and powerful stereotypes that link mental illness and violence (6).

## That photograph

The photograph was taken when Clunis was being driven away from the sentencing hearing. At this point, he had been in custody for some time and receiving treatment. He appears heavily sedated and significantly overweight – a well-documented side effect of major tranquilisers. The photograph was often used alongside a photograph of Jonathan and Jayne Zito. I am clearly not being critical of the family who suffered unimaginable pain and loss. It is the way that the media use the photographs to create a series of binary oppositions. The Clunis case became a reference point and representation of the failings of community care in the late 1980s and early 1990s in the UK. The case and the photograph were used in numerous newspaper articles and features. The photograph and accompanying text rarely, if at all, provides any further context. For example, details of Clunis’ personal and family history or the attempts they made to support him are barely mentioned in any accounts of the case (7).

## Media reporting and crime

The Clunis case took place before the vast explosion of 24-hour rolling news and the development of social media that have fundamentally changed reporting and citizens consumption of news. At the time of these events, newspapers, TV, and radio news dominated the reporting landscape. Crime and penal policy became a key feature in the creation of the authoritarian populism that developed from the mid-1970s onwards (8, 9). Politicians such as Regan and Thatcher in establishing new political coalitions made calls to voters over the heads of elites of academics and liberal penal policy makers portraying them as weak on crime and too concerned with the rights of offenders (10).

Only around 40% of homicides are reported in national newspapers. An analysis of journalists’ decision making, revealed that homicides that take place in London are more likely to get media coverage because of the concentration of news organisations in the city. Alongside this, factors such as the age, gender, race, and social status of both the perpetrator and victim play a role in the amount of coverage that a case will receive. A motiveless, stranger attack like this one with a victim such as Jonathan Zito will receive significant coverage. This can be contrasted with cases, for example, of domestic homicide (11, 12).

Moral panics are a manifestation of underlying social and political disquiet (13). There are certain areas, for example, youth culture including the emergence of new forms of music and subcultures, crime including the use of drugs where moral panics occur at regular intervals. Hall et al. suggest that the panic is triggered by an event. They describe the ways in which these events cause *‘public disquiet* (13)*’.* The response to this panic not only includes societal control mechanisms, such as the courts, but the media also becomes an important mediating agency between the state and the formation of public opinion. Concerns in these areas focus on crime as manifestations of wider social concerns (13). Racialised representations of crime became ciphers for wider societal tensions. They serve the joint purposes of recreating and reinforcing racist ideas but also representing issues in ways that divert from the underlying structural causes (14, 15). The term ‘*conjuncture*’ captures the fluidity of modern political, economic, and cultural life. The term also requires any analysis of a political and social issues to consider a much broader range of factors including media representations (16, 17).

The moral panic surrounding the policy of community care panic of the early 1990s was triggered by homicides that were conducted by individuals who had a history of mental illness and contact with mental health services. The crisis in community based mental health services in England and Wales in the late 1980s and early 1990s came to be symbolised by one image - a photograph of a Christopher Clunis. A random violent attack encapsulates one of the major concerns of late modernity – the fear of violent crime. The fact that the attacker was a homeless mentally ill man unknown to Zito heightened the fears. This case link mental health, race, and crime. The folk devil of the urban crime moral panic of the early 1970s was a Black youth (13). The racist stereotype of young Black men as violent urban criminals is a deeply entrenched urban folk devil. In this period, this figure became a “mugger” – someone who commits street robberies. The folk devil has been reconstituted in later moral panics about rap music or gangs.

The photograph of Clunis is not a formal police “mugshot” but fulfils a similar role. The mugshot has become a powerful and instantly recognisable construction of the other (16). It has a key role in the modern rituals and spectacle of crime and punishment (17). In the modern media saturated world, we do not have to be present to take part in the spectacle. News photographs noted that they are powerful as they are presented a visual representation of the “real world.” In this process of representation, the ideological underpinnings and meanings of the image are repressed (18). In this case, the photograph was playing on long standing tropes that associate race and mental illness with violence (19). There are two elements to the process of signification in the news photograph. The first is the news value of the story. The second is the photograph as an ideological sign. The photograph can be seen as a way of indexing ideological themes (20). The use of photographic images is a way of removing the structural, social, and political context of an event or crime 18). In this case, the representation of Clunis is a way of isolating him from the wider institutional context whilst referring back to long standing racist tropes.

## Discussion

The experiences of young Black men with mental health services and the Criminal Justice System (CJS) cannot be separated from wider experiences of poverty, racism, and marginalisation. However, within mental health services, there is a significant body of research that shows young Black experiences are coercive and punitive rather than therapeutic experiences (21, 22). This body of research, much of which took place, around the time that Clunis was living in the community that demonstrated the impact of racist stereotyping. Young Black men were and are more likely to come into mental health services via the CJS. They are more likely to be seen as “dangerous” and less likely to be offered counselling or other alternatives to a medicalised approach to mental health crisis Service users regard services as a threat rather than a potential source of support. These barriers mean that crisis interventions are more likely. This is a long-standing issue that has not been tackled despite a series of policy and other initiatives. Black in patients experienced admission as “*a racist and racialized experience, inseparable from a wider context of systemic racism and inequality”* (23).

Deeply entrenched cultural representations of mental illness and the mentally ill, generate stigma and fear. These representations may change and develop but the core elements remain. These include an association with mental illness and violent behaviour. In her essay *In Plato’s Cave*, Sontag (24) describes photography as a means of creating and maintaining nostalgia. She notes that even when photographs appear or claim to reflect a social reality, there are, in fact, social constructs. The photograph is manipulated in various ways to produce a desired effect. Documentary or news photographs are presented as documenting a reality, but they do more than capture the reality of an event. This photograph echoes a number of racial stereotypes of young Black men – for example, the emphasis on size and the link with violence. Sontag (25) argued that “*The Western memory museum is now mostly a visual one’.* In the twenty years since, this trend has intensified. In her discussion of the photographs of the torture of prisoners at Abu Ghraib noted the power of photographs to shape our memory and understanding of events and individuals.

## Outlook

Following deinstitutionalisation, representations of mental illness in popular culture and other discourses shifted. This shift saw the image of the psychiatric patient became racialised and increasingly associated with violence and aggression (26). In 1994, *The Prins Inquiry* (27) examined the death of a Black patient, Orville Blackwood at a secure hospital in England. The Inquiry found that nursing staff openly used the phrase “*big, black and dangerous”* to describe him and other patients. This encapsulates a series of racist stereotypes that are deeply embedded within mental health services and the wider society. The photograph of Christopher Clunis can be viewed as a mediated representation of this phrase.

## Data availability statement

The original contributions presented in the study are included in the article/supplementary material. Further inquiries can be directed to the corresponding author.

## Author contributions

IC: Writing – original draft.
